# Development of resident and migratory three-spined stickleback, *Gasterosteus aculeatus*

**DOI:** 10.1371/journal.pone.0295485

**Published:** 2024-07-18

**Authors:** Megan Barnes, Lisa Chakrabarti, Andrew D. C. MacColl

**Affiliations:** 1 School of Life Sciences, University of Nottingham, Nottingham, United Kingdom; 2 School of Veterinary Medicine and Science, Sutton Bonington Campus, University of Nottingham, Loughborough, United Kingdom; 3 Medical Research Council Versus Arthritis Centre for Musculoskeletal Ageing Research, Nottingham, United Kingdom; University of Iceland, ICELAND

## Abstract

The three-spined stickleback (*Gasterosteus aculeatus*) is a teleost fish and a model organism in evolutionary ecology, useful for both laboratory and natural experiments. It is especially valued for the substantial intraspecific variation in morphology, behaviour and genetics. Classic work of Swarup (1958) has described the development in the laboratory of embryos from a single freshwater population, but this was carried out at higher temperature than many stickleback would encounter in the wild and variation between populations was not addressed. Here we describe the development of embryos from two sympatric, saltwater ecotypes of stickleback from North Uist, Scotland raised at 14°C, the approximate temperature of North Uist lochs in the breeding season. The two ecotypes were (a) a large, migratory form in which the adults are completely plated with bony armour and (b) a smaller, low-plated form that is resident year-round in saltwater lagoons. By monitoring embryos every 24-hours post fertilisation, important characteristics of development were observed and photographed to provide a reference for North Uist ecotypes at this temperature. Hatching success was greater than 85% and did not differ between resident and migratory stickleback, but migratory eggs hatched significantly earlier than the resident ecotype. Our work provides a framework that can now be used to compare stickleback populations that may also grow in distinct environmental conditions, to help understand the breadth of normal developmental features and to characterise abnormal development.

## Introduction

The three-spined stickleback (*Gasterosteus aculeatus*) has been increasingly studied and raised in aquaria [1]. The repeated adaptation of oceanic stickleback to freshwater make it an attractive model to investigate parallel evolution [2]. As such, the three-spined stickleback has become an important model in evolutionary genomics, with both laboratory and natural experiments widely reported. Despite much recent research on variation in the morphology, behaviour and genomics of stickleback, there is a paucity of work describing variation in the development of stickleback from fertilisation to hatching. Swarup 1958 described in detail the key stages in development of the three-spined stickleback, building upon Vrat, 1949 [3] and Kuntz & Radcliffe, 1917 [4]. However, Swarup’s embryos developed in the lab at 18–19°C, a relatively high temperature for stickleback, and did not consider the possibility of variation between populations.

Temperature is known to greatly influence development time in fish [5–7], so it is likely that at a colder, more physiologically relevant temperature for many stickleback populations, development time will be increased but this has not been quantified in the stickleback. A large amount of stickleback research is conducted on populations from North Uist (Western Isles, Scotland) [8–10]. Water temperatures in the island’s lagoons have been recorded as between 13.1 and 15.0°C during the breeding season (May 2022 and 2023), a considerably lower temperature than stickleback development has previously been assessed at [11]. Optimum growth for the stickleback has been recorded at 21°C [12]. It is therefore likely that the colder temperatures experienced by stickleback in more northern regions, such as on North Uist, will alter both growth and development. Given the wide distribution of stickleback across the Northern Hemisphere encompassing large variation in environmental variables and therefore stickleback morphology, it is useful to quantify development of the stickleback at lower temperatures, and to compare contrasting ecotypes.

On North Uist, there are two morphologically distinct ecotypes that occur sympatrically in saltwater: migratory and lagoon resident [10]. Migratory stickleback are large and completely plated, spending most of their lives at sea but migrating to brackish water to spawn, whereas smaller, low plated lagoon resident fish live permanently in coastal lagoons. Resident stickleback lay smaller clutches than migratory [13] and as previous work hints at a negative relationship between clutch size and egg volume [14], migratory fish likely lay smaller eggs. High levels of reproductive isolation are maintained between the ecotypes, with an estimated hybridisation rate of ~1% [10]. Migratory and lagoon resident ecotypes vary in both morphology and genetics [10], so there are likely also differences in egg size and developmental timing, as found between Arctic charr morphs [15].

Here, we compare the development of migratory and resident stickleback from North Uist at a temperature naturally experienced by these populations, and provide reference photographs for future work.

## Methods

To compare development in sympatric stickleback ecotypes at a physiologically relevant temperature (14°C) and provide a photographic resource for future studies, we monitored the development of lagoon resident and migratory stickleback from North Uist, starting at fertilisation through to hatching. Coloured photographs of resident stickleback were taken to record the key stages and any differences between these and the migratory embryos were assessed. Hatching success and time to hatching were also recorded for both ecotypes. All work was carried out according to UK Home Office regulations (the Animal Scientific Procedures Act 1986 [16] and ensuing legislation), under Project Licence (PP5421721) held by Andrew MacColl. This was approved by the University of Nottingham Animal Welfare and Ethics Review Board.

Fieldwork was conducted on North Uist between 30^th^ April and 19^th^ May 2023. To collect breeding wild stickleback of both sexes and ecotypes, mesh traps were left in Loch an Duin (57.64245, -7.209207), a saltwater lagoon in the North-East of the island, for 24-hours. Migratory and lagoon resident crosses were made, following standard procedures [17], by squeezing eggs from gravid, euthanised females into small petri dishes and mixing them with testes from euthanised reproductive males. Fish were euthanised with an overdose of tricaine methanesulfonate (400mg/L) followed by destruction of the brain in accordance with Schedule One of UK Home Office regulations. After fertilisation, eggs were covered in sterile water which was the media used throughout egg development. Egg number per clutch was kept between 20 and 30 where possible. Six crosses per ecotype were made using different males and females for each cross, and each was raised in a separate petri dish. Embryos were maintained at 14°C, the approximate water temperature of North Uist lochs in April, in an incubator (ICT-P Falc, portable mini incubator) where air could fully circulate. Every ~24-hours post-fertilisation, petri dishes were briefly taken out of the incubator and media carefully removed with a Pasteur pipette and then replaced with fresh, temperature-matched media. Other than changing the media and any turning of the eggs as a consequence of viewing them under the microscope, no additional egg care was implemented. Each dish was observed under a dissecting microscope (Olympus SZ61), key features of developing embryos noted and photographs taken. Embryo development was also scored based on Swarup 1958. The same person (MB) scored each cross throughout the experiment. This was repeated daily, until two days after the first hatched stickleback larvae was observed in each clutch.

Hatching success was calculated for each clutch of migratory and lagoon resident ecotypes separately as percentage of successfully hatched larvae. The number of days until the first appearance of a fully hatched stickleback and the number of days until 50% of the clutch had hatched was noted. Hatching success was compared between ecotypes using a binomial GLM with logit link. In one migratory clutch, 17 unfertilised eggs were observed 48-hours post-fertilisation; this was likely due to hardening of the eggs in the female reproductive tract which can be common in stickleback [18]. In this case hatching success was recalculated as the number of successfully hatched stickleback per total number of fertilised eggs, and this value was used in the model. Time to hatch (days) was compared between migratory and lagoon resident stickleback using two-sample Wilcoxon rank sum tests. To assess differences in development between migratory and lagoon resident stickleback, the score based on Swarup’s 1958 taxonomy was plotted at each 24-hour timepoint for both ecotypes. We tested for differences between ecotypes in the development score over multiple days using two-sample Wilcoxon rank sum tests, and used the false discovery rate (FDR) method to control for type 1 errors [19]. Considering the small number of tests and clutches used in this analysis, we set a relatively relaxed FDR threshold of 0.1, which allowed for 10% of significant results to be false positives. As days to 50% hatched and observable differences in development were also assessed between ecotypes, we could be confident that differences in hatching time between migratory and resident stickleback was not a type I error [20].

As egg size could in part explain any differences in hatching success or days to hatch between migratory and resident stickleback, we have included measurements of egg volume for the two ecotypes found on North Uist, from two different lochs sampled in 2007 and 2011. Migratory and resident stickleback were collected from the saltwater lochs Ob nan Stearnain (57.601667, -7.172778) and Fairy Knoll (57.635278, -7.215000). Eggs were assumed to be ellipsoid and were measured using a dissecting microscope with an eyepiece graticule. Length (largest dimension when viewed from above) was measured and the measurement perpendicular to this was assumed to be the diameter at the widest point. These measurements were halved to get corresponding half-axis measurements, a and b respectively. These values were used to calculate volume for between 5 and 10 eggs per mature female, as 4/3πab^2^. A mean egg volume was then calculated for each female and this was compared between ecotypes using a linear mixed effects model with ecotype as a fixed effect and loch as a random effect.

All analyses were done using the R language and performed in R Studio [20] and an alpha value of 0.05 used for statistical tests.

## Results

**Fig 1:**
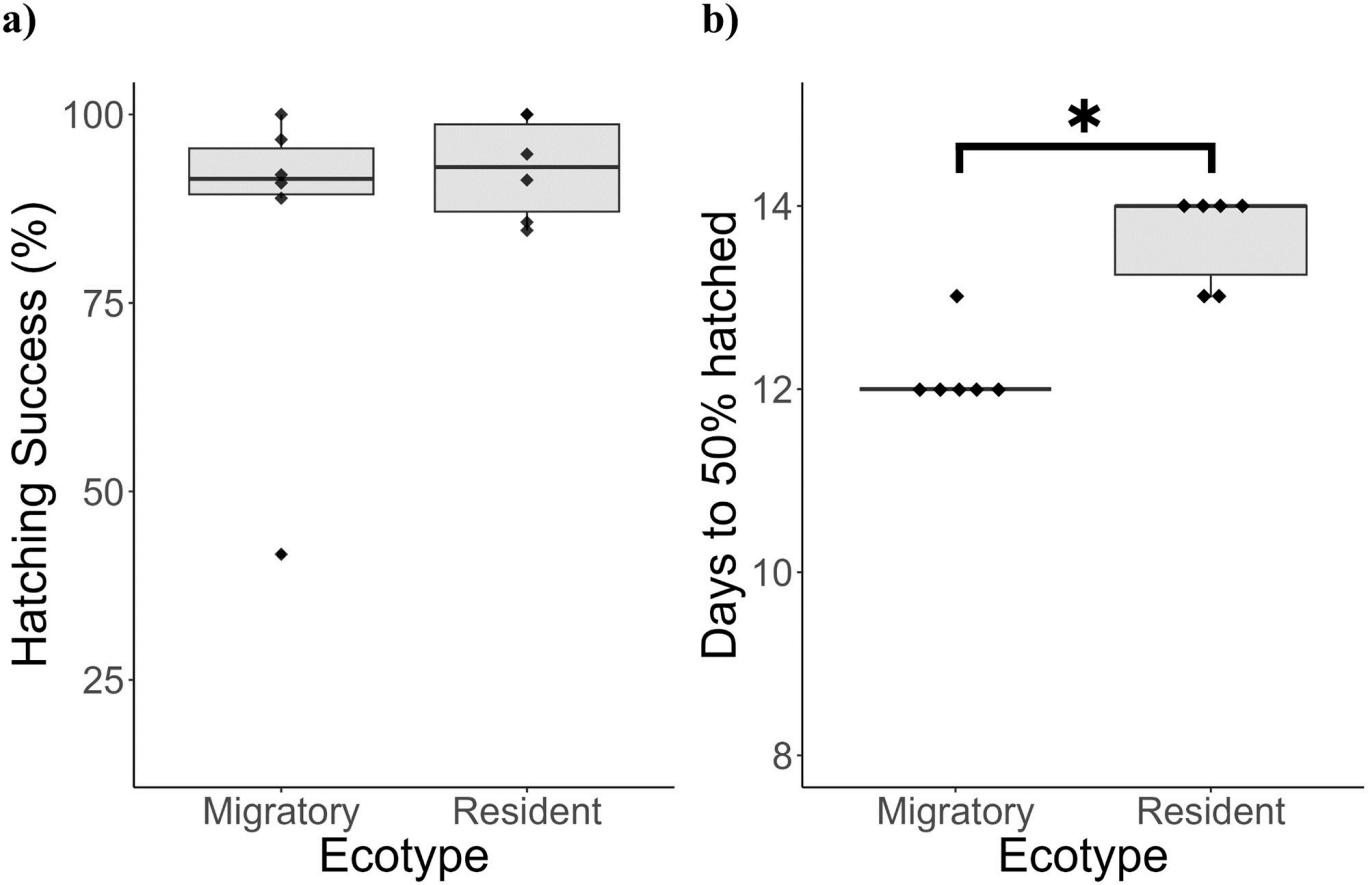
Hatching success (a) and days to 50% hatched (b) for migratory and resident three-spined stickleback incubated at 14°C. Hatching success was calculated per clutch as number of successfully hatched larvae divided by initial number of eggs. Days to hatch is calculated from fertilisation (day 0) until 50% of the clutch had hatched. Boxplot shows median and interquartile range and all data values are displayed as points. n = 6 clutches per ecotype. Significant differences, p < 0.05, marked with an asterisk.

**Fig 2:**
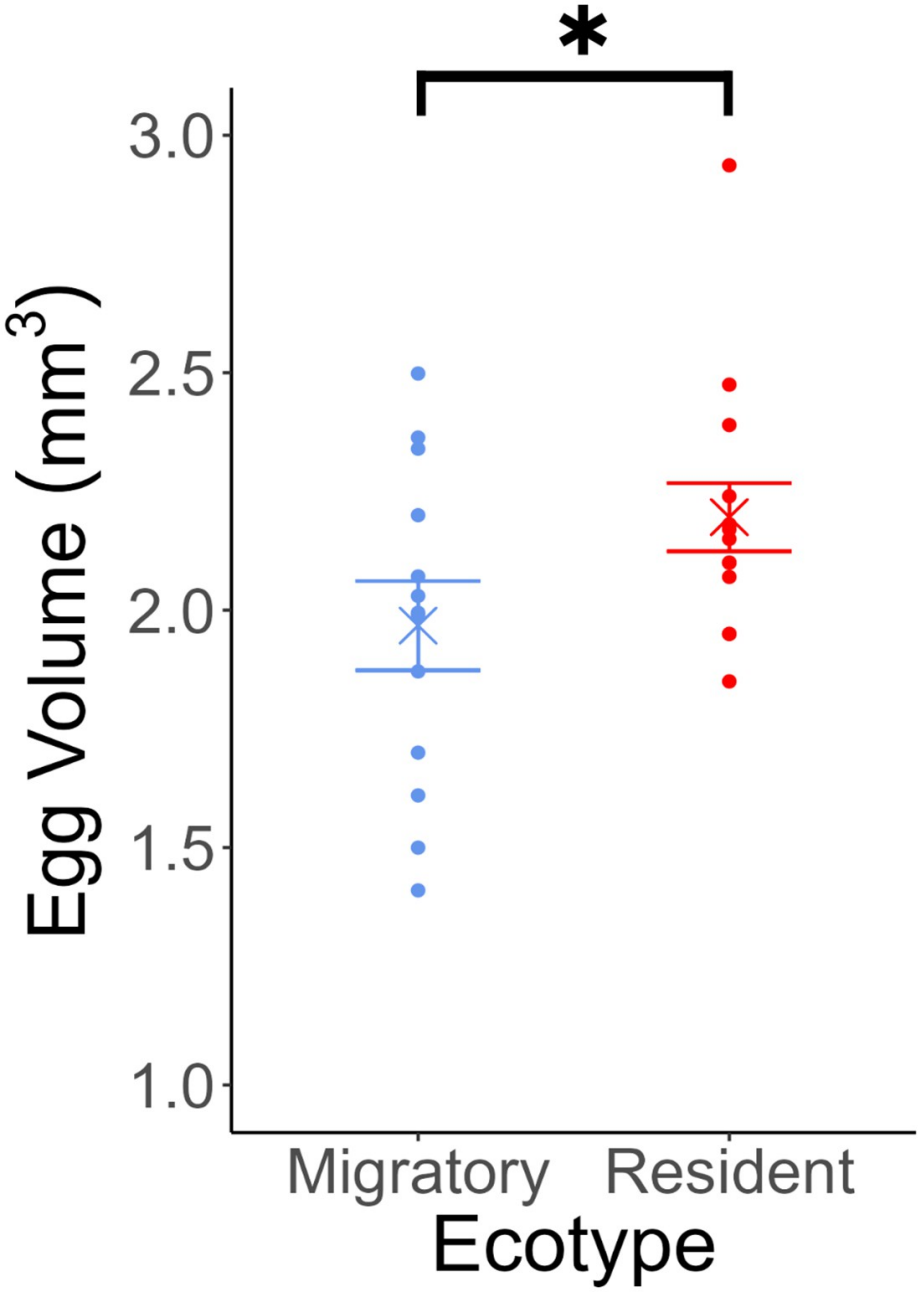
Egg volume for migratory and resident three-spined stickleback. Mean value for each ecotype is shown with a cross and error bars show standard error. Mean egg volumes for each female are displayed as points; n = 27. Significant differences, p < 0.05, marked with an asterisk.

The number of eggs selected per clutch averaged 23.6, with means of 27.0 and 20.2 eggs for migratory and resident clutches respectively (in total, clutches are larger than this, especially for migratory fish). There was no difference in hatching success between ecotypes (Wald X^2^_1_ = 0.673, P = 0.412; Table 1, Fig 1a), with hatching success averaging 85.0% in migratory stickleback and 92.7% in lagoon resident fish (Fig 1a). The number of days until appearance of the first hatched larvae and the number of days until 50% of the clutch had hatched were significantly earlier in migratory than lagoon resident stickleback (Wilcoxon rank sum test: First larvae: p = 0.00300; 50% hatched: p = 0.00488; Table 1, Fig 1b). The first migratory fish hatched after 11.7 days on average and 50% of the clutch hatched after 12.2 days (clutches hatched on days 11 or 12) and the first resident fish hatched after 13.2 days, with 50% of the clutch having hatched after 13.7 days (clutches hatched on day 13 or 14). Resident stickleback had significantly larger eggs than migratory (χ^2^ = 3.84, df = 1, p = 0.0499; Table 1, Fig 2).

**Table 1:** Hatching success, days to hatch and egg volume.

	Migratory	Resident
Hatching Success (%): mean +/- SD	85.0 +/- 21.6	92.7 +/- 6.7
Day to first hatched stickleback: mean +/- SD	11.7 +/- 0.5	13.2 +/- 0.5
Day to 50% hatched stickleback: mean +/- SD	12.2 +/- 0.4	13.7 +/- 0.4
Egg Volume (mm^3^): mean +/- SD	1.97 +/- 0.34	2.20 +/- 0.27

**Fig 3:**
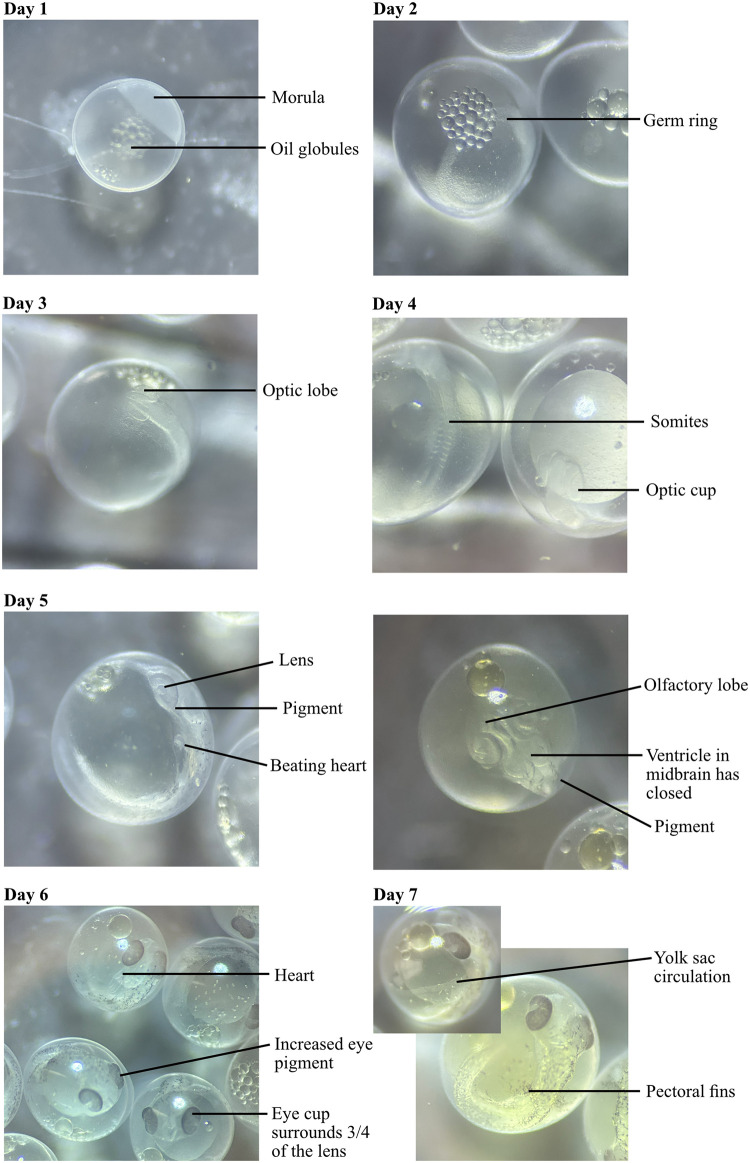
Three-spined stickleback development from day 1 (24-hours post fertilisation) to day 7. Images taken using a dissection microscope with a dark background on the stage, for contrast. Key features are labelled.

**Fig 4:**
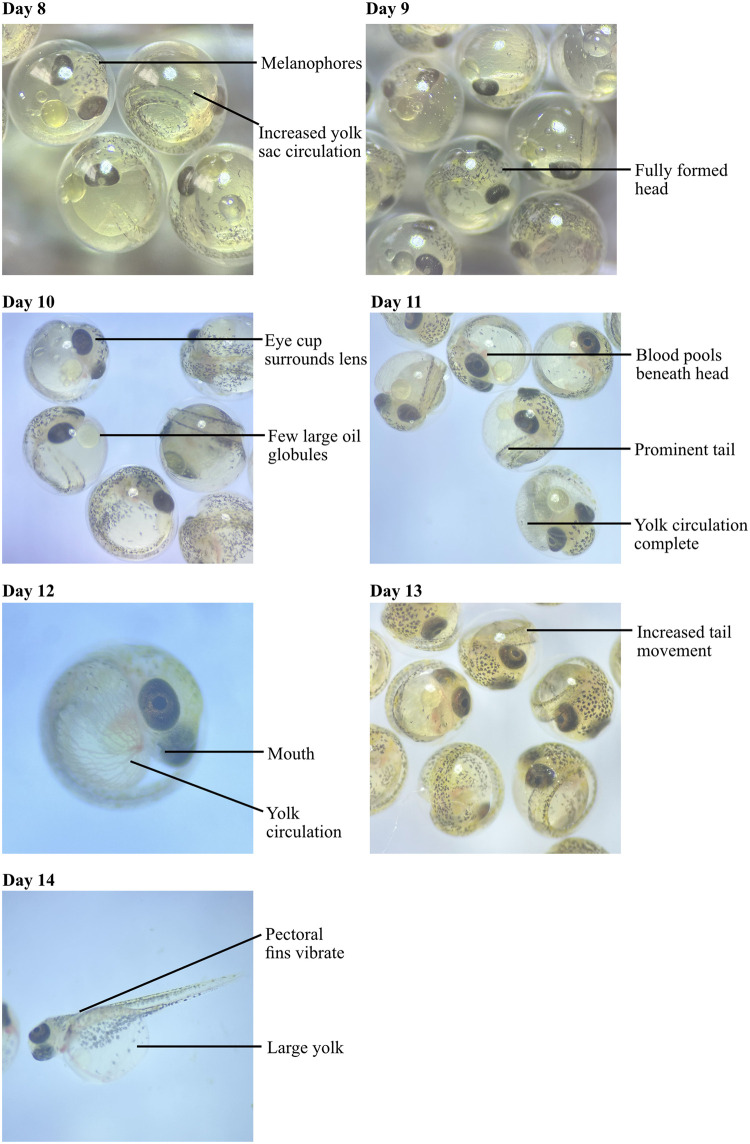
Three-spined stickleback development from day 8 up until hatching. Images taken using a dissection microscope with either a dark or light background on the stage, for contrast. Key features are labelled.

Although there were quantitative differences in developmental stage between the ecotypes at days 2 and 12 (see below), our observations of the clutches revealed that development was very similar in migratory and resident clutches. Therefore, we only describe the development of lagoon resident clutches in detail, at each 24 hour interval. Day 0 is the day of fertilisation, day 1 refers to 24-hours post fertilisation, and so on. Figs 3 and 4 show a photo time-series of development which gives a clearer impression than the line drawings that were previously available.

*Day 1*: By 24-hours post fertilisation, the egg had gone through the 2, 4, 8, 16 and 32-cell stage, reaching stage 9 in the Swarup series. The cells divided further, becoming smaller; these form the morula which sits on the yolk. Oil globules were visible in the yolk.

*Day 2*: Gastrulation had begun. A germ ring formed. The more opaque ring of cells seen in Swarup stage 12 were often not visible until the eggs were gently rotated, as the germ ring was usually on the ventral side. A dark background aided visualisation of features early in development.

*Day 3*: Optic lobes had formed either side of the forebrain (Swarup stage 16). The embryo protruded from the surface of the yolk sac.

*Day 4*: Somites appeared in the middle of the embryo. Rather than from the side of the embryo, as shown in Swarup 1958 stage 17, somites were best seen by gradually focusing through the yolk sac to the opposite side (Fig 3, day 4). Around six or seven pairs were clear in most cases, although occasionally there were more. The head had differentiated so that the optic lobes became optic vesicles where central cavities were visible, and then optic cups as lenses formed. The brain developed further so that separation between the mid- and hindbrain was clear.

*Day 5*: The heartbeat was apparent on the left side of the embryo (Swarup stage 19). This was most clear to see when looking at the embryo from the side, where only one eye is visible (Fig 3, day 5). Some pigment began to appear, starting on the outer margins of the eye and some scattered areas on the body. Ventricles in the midbrain had closed. Some gentle rotation of the eggs was necessary to see both the heartbeat and the features of the head, hence two images are provided for day 5 in Fig 3.

*Day 6*: More eye and body pigment was present so that most of the eye cup was dark in colour and this now surrounded almost all of the lens except for the lower portion. The tail occasionally moved, although at this point it was infrequent and difficult to capture. The split in the hindbrain could no longer be seen. The heart was larger and the three chambers were visible.

*Day 7*: Yolk sac circulation could be seen on the left of the embryo; often gently rotating the embryo was necessary to identify this. Pectoral fins started to develop. There was more pigment, including on the yolk sac. The head of the embryo became shorter and broader (Swarup stage 22).

*Day 8*: More melanophores were visible on the head. The yolk sac circulation increased in area; at this stage this was difficult to see as the colour is similar to the yolk sac so switching between a dark and light background helped distinguish this. Tail movement became more frequent and the eye pigment was darker throughout the eye cup. The split in the forebrain was still visible.

*Day 9*: The ventricle of the forebrain closed so the head was fully formed. The yolk sac circulation was almost complete (Swarup stage 23).

*Day 10*: The eye cup now completely surrounded the lens, which became dark in colour. The eye cup surrounding the lens happened at an earlier stage than in Swarup’s descriptions as here this occurs prior to the completion of the yolk sac circulation and formation of the mouth, whereas this is noted at stage 24 of Swarup’s descriptions where the embryo is almost ready to hatch (the final stage, approx. 24 hours prior to hatching). There were only a few larger oil globules and these were situated in front of the head. Blood also collected in front of the head. A change to a lighter background at this point in development was found to increase contrast between the pigmented features and the background, making identification easier.

*Day 11*: The blood collecting in front of the head became more apparent as the yolk circulation was complete and its red colour is clear, especially on a light background. The mouth formed and the tail became more prominent. To observe the mouth, gently moving the embryos was necessary to position the head of the embryo in the best light to capture the mouth; it was best observed looking at the front of the embryo (as in Fig 4, day 12).

*Day 12*: The amount of pigment on the body increased further. Tail movement was more frequent and the pectoral fins vibrated. The embryos displayed all key features described in stage 24 of Swarup’s taxonomy and were ready for hatching.

*Day 13 or 14*: At day 13 or 14 hatching commenced. The head of the embryo pushed against the shell of the egg and broke free. Tail movement then freed the rest of the embryo. The hatched embryo was transparent and lay on its side as the yolk is large. The head remained curved around the edge of the yolk.

**Fig 5:**
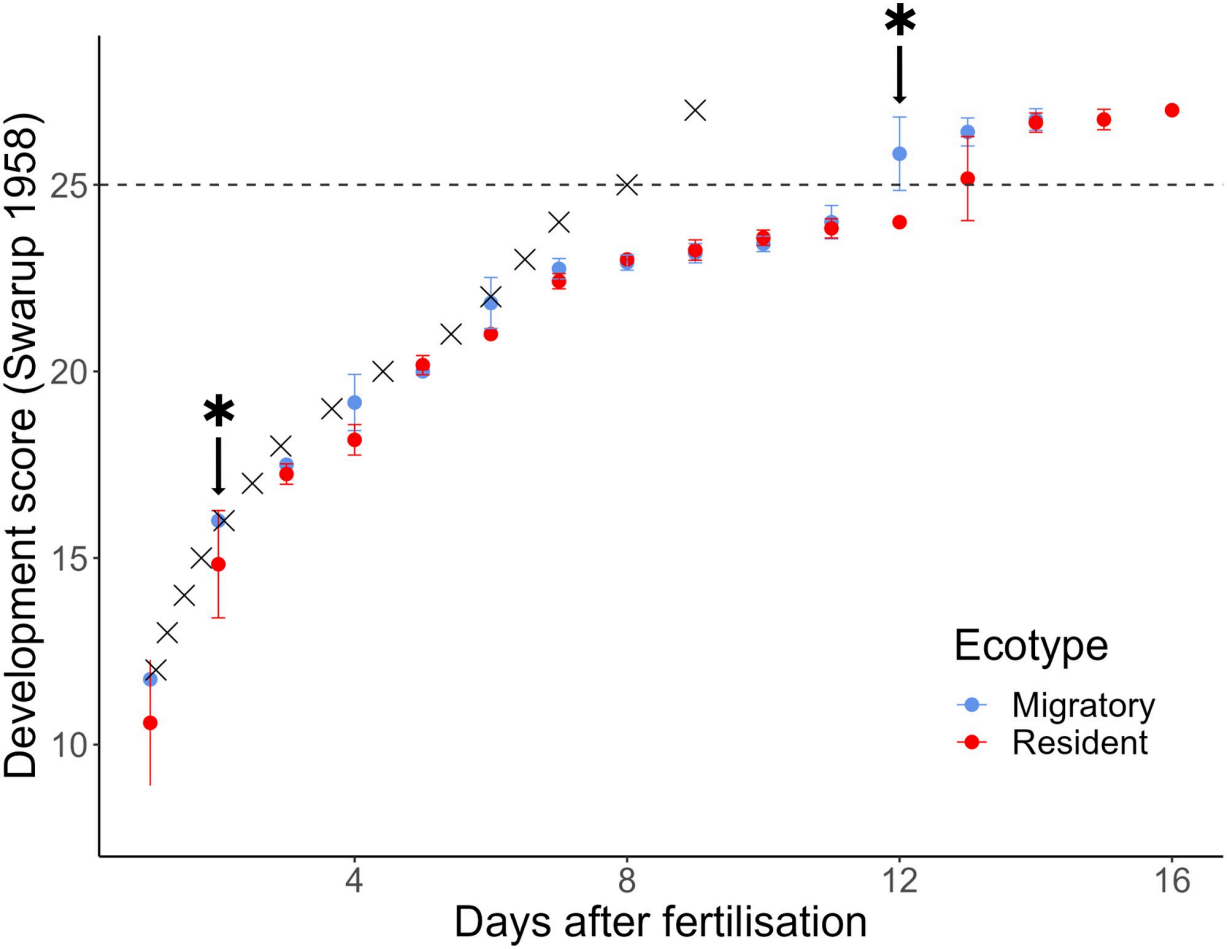
Development of stickleback embryos through time (days after fertilisation) estimated as the Swarup (1958) score. Migratory stickleback clutches are shown in blue and residents in red with error bars showing standard deviation. Clutches were raised at 14°C. Grey crosses show the development score from Swarup 1958 for comparison, where stickleback were raised at 18–19°C. Dotted line shows when stickleback have hatched. Asterisk shows days where there was a significant difference (p < 0.05) between migratory and resident scores (two-sample Wilcoxon rank sum tests) that remained significant after correcting for multiple comparisons using the FDR method: FDR = 0.10.

There were differences in development between lagoon resident and migratory stickleback: comparing development each day, migratory clutches were at a significantly higher score than residents at days 2, 4, 6 and 12 (Fig 5, Table 2), but after correcting for multiple testing only differences at days 2 and 12 remained significant (Table 2). Differences were therefore early in development when migratory clutches had a higher score and were therefore more developed than residents, and late in development (day 12) when the eggs of residents took longer to hatch than migratory clutches after reaching the final stage (24) in Swarup’s scoring (Fig 5).

**Table 2:** Wilcoxon rank sum test results for comparisons between migratory and resident stickleback development scores at each day ranked in order of increasing p value, with multiple comparisons testing of results. FDR set to 0.10.

Day Tested	P value (Wilcoxon Rank Sum Test)	Rank	Benjamini-Hochberg significance
12	0.008113	1	Significant
2	0.009310	2	Significant
6	0.02766	3	Not significant
4	0.02889	4	Not significant
7	0.05429	5	Not significant
3	0.07053	6	Not significant
13	0.07955	7	Not significant
5	0.1739	8	Not significant
10	0.2184	9	Not significant
1	0.2361	10	Not significant
8	0.4047	11	Not significant
11	0.5407	12	Not significant
9	0.6404	13	Not significant
14	0.7077	14	Not significant

Progression of development in our clutches of migratory and resident stickleback (raised at 14°C) also differed strongly from those of Swarup raised at 18–19°C. Early in development (days 1 to 6) there is only a small difference, with those raised at 18–19°C having, in general, a slightly higher development score (Fig 5). However, by day 7 there is a large difference in score, with Swarup’s having reached stage 24, the final stage before hatching, but both resident and migratory clutches raised at 14°C averaging a score of 22.6 (migratory mean was 22.8 and resident 22.4). Those raised at the higher temperatures hatch (stage 25) at days 6 to 8, while clutches raised at the lower temperature remain at the later stages of development (stages 22 to 24) for a longer time, before hatching between days 11 and 14.

## Discussion

We describe visual features of development in the embryos of two contrasting ecotypes of three-spined stickleback, every 24 hours post-fertilisation, with key structures highlighted in photographs to increase the ease of identification of important features. This builds upon the work of Swarup (1958), where stages were named after key morphological characteristics and described in detail in stickleback embryos raised at 18 to 19°C. However, as stickleback development is strongly influenced by temperature, this work assessed these key features daily in stickleback developing at a lower, physiologically relevant temperature for where these populations would be found naturally, and to compare the two ecotypes. The hatching success of both ecotypes was high, with no differences between them. We have presented only coloured images of resident stickleback because, aside from the difference in hatching time, qualitatively we did not observe any differences in development between ecotypes. As we viewed embryos every 24 hours it remains possible that finer differences in detail exist but could not be observed here.

Migratory clutches tended to be further developed than residents from days 1 to 7, although this only reached significance at day 2. From day 8, no difference in development stage was observed between ecotypes, so resident clutches had ‘caught up’ with migratory, and both ecotypes had reached stage 24 at the same time. Migratory clutches then hatched on day 11 or 12 while residents remained at the last stage prior to hatching. The eggs of resident stickleback therefore hatched on average 1.5 days later than those of migratory fish. As embryos were checked every 24-hours, the hatch day was recorded as the day where 50% or more of the clutch had hatched. This could therefore be slightly longer than actual time to hatching (as stickleback may have hatched sometime within the day prior), but this should not cause a systematic bias between the ecotypes. Indeed, the difference between ecotypes was large, with little overlap in hatch day between groups.

The migratory and resident forms are known to vary greatly in morphology and genetics, so a difference in development is not unexpected [10], and diverged Arctic charr morphs have been found to vary in egg size, development timing and size at hatching [15]. Migratory stickleback have larger clutches than resident [13] and there is a trade-off between clutch size and egg size [13, 14], suggesting that the eggs of migratory fish are likely smaller than those of residents. Indeed, we show quantitatively that this is the case for North Uist populations, with resident eggs having a larger volume than migratory eggs. The decreased yolk size and therefore nutrients for migratory embryos may explain the reduced time to hatch, as well as additional differences in the egg or membrane itself. The finding that migratory clutches with smaller eggs hatched earlier than residents with larger eggs agrees with previous work compiling development times from 84 species of teleost fish; they found that smaller eggs developed faster than larger eggs when all other factors, including temperature, were controlled [7]. Further studies on the egg of both ecotypes would be required to fully understand these findings. Temperature, pH and dissolved oxygen have also previously been implicated in stickleback growth and development [12, 21, 22], but as rearing conditions were consistent here, these cannot explain the differences in development between ecotypes.

In addition to the ecotype differences observed, by comparing resident and migratory stickleback raised at 14°C to stickleback raised at 18–19°C by Swarup (1958), the effect of incubation temperature can be explored, although this is limited as Swarup provides only an estimate for the time to reach each stage. All stickleback were raised in freshwater but different populations were used between studies, so additional population effects can also not be ruled out. Time to hatch was greater than 3 days longer at 14°C than Swarup found at the warmer temperature of 18–19°C. This reduced hatching time at increased temperatures has been reported across many fish species [5, 7], with hatching time often inversely proportional to incubation temperature, for example in Haddock (*Melanogrammus aeglefinus*) [6]. As differences between temperatures were not apparent until seven days post fertilisation, temperature has limited influence early on in stickleback development, up until yolk sac circulation, but warmer temperatures then induce hatching earlier. Similar findings have been made in the zebrafish (*Danio rerio*), where the same rate of early development was observed at a wide range of temperatures [23]. In the three-spined stickleback, temperature greatly effects paternal care behaviour, reproductive success and growth rate post-hatching [12, 24], but effects on embryo development have rarely been studied.

This study has revisualised the key characteristics in three-spined stickleback development up until hatching, following the landmark work of Swarup (1958). Using North Uist lagoon resident and migratory populations that live in sympatry during the breeding season provides further information on how different ecotypes develop at a temperature they would naturally experience. It provides a set of features that are clear at each day post fertilisation to allow comparison between future treatments, particularly important if we want to discover how stickleback embryos will cope with the increasing water temperature and carbon dioxide levels that may be experienced. These stages also provide a baseline for any abnormalities in development to be compared to. A high yet consistent hatching success between both ecotypes has also been shown and that migratory stickleback hatch at least a day earlier on average than resident stickleback from the same loch. Further investigation into this in natural populations, and studies of migratory and resident stickleback eggs, would be necessary to explain this pattern. However, as this species is often raised in aquaria at the temperature used here, this information adds to our knowledge of how long stickleback incubation lasts before hatching.

## Supporting information

S1 Dataset Clutch information, development score based on Swarup (1958) and egg size data. (XLSX)
